# Senescent CD4^+^CD28^−^ T Lymphocytes as a Potential Driver of Th17/Treg Imbalance and Alveolar Bone Resorption during Periodontitis

**DOI:** 10.3390/ijms23052543

**Published:** 2022-02-25

**Authors:** Luis González-Osuna, Alfredo Sierra-Cristancho, Emilio A. Cafferata, Samanta Melgar-Rodríguez, Carolina Rojas, Paola Carvajal, Cristian Cortez, Rolando Vernal

**Affiliations:** 1 Periodontal Biology Laboratory, Faculty of Dentistry, Universidad de Chile, Santiago 8380492, Chile; asierra@odontologia.uchile.cl (A.S.-C.); ecafferata@cientifica.edu.pe (E.A.C.); smelgar@odontologia.uchile.cl (S.M.-R.); carolina.rojas.p@ug.uchile.cl (C.R.); 2 Faculty of Dentistry, Universidad Andres Bello, Santiago 8370035, Chile; 3 Department of Periodontology, School of Dentistry, Universidad Científica del Sur, Lima 15067, Peru; 4 Department of Conservative Dentistry, Faculty of Dentistry, Universidad de Chile, Santiago 8380492, Chile; pcarvajal@odontologia.uchile.cl; 5 Center for Genomics and Bioinformatics, Faculty of Sciences, Universidad Mayor, Santiago 8580745, Chile; cristiancortezplaza@gmail.com

**Keywords:** cell senescence, T-lymphocytes, CD28, periodontitis, alveolar bone loss, Th17 lymphocytes, Tregs

## Abstract

Senescent cells express a senescence-associated secretory phenotype (SASP) with a pro-inflammatory bias, which contributes to the chronicity of inflammation. During chronic inflammatory diseases, infiltrating CD4^+^ T lymphocytes can undergo cellular senescence and arrest the surface expression of CD28, have a response biased towards T-helper type-17 (Th17) of immunity, and show a remarkable ability to induce osteoclastogenesis. As a cellular counterpart, T regulatory lymphocytes (Tregs) can also undergo cellular senescence, and CD28^−^ Tregs are able to express an SASP secretome, thus severely altering their immunosuppressive capacities. During periodontitis, the persistent microbial challenge and chronic inflammation favor the induction of cellular senescence. Therefore, senescence of Th17 and Treg lymphocytes could contribute to Th17/Treg imbalance and favor the tooth-supporting alveolar bone loss characteristic of the disease. In the present review, we describe the concept of cellular senescence; particularly, the one produced during chronic inflammation and persistent microbial antigen challenge. In addition, we detail the different markers used to identify senescent cells, proposing those specific to senescent T lymphocytes that can be used for periodontal research purposes. Finally, we discuss the existing literature that allows us to suggest the potential pathogenic role of senescent CD4^+^CD28^−^ T lymphocytes in periodontitis.

## 1. Introduction

Periodontitis is the most prevalent osteolytic disease in humans in which factors directly related to aging significantly contribute to the development of the disease [1]. During periodontitis, chronic inflammation in response to bacterial challenge leads to irreversible resorption of tooth-supporting alveolar bone and finally, tooth loss [1,2]. Indeed, the development of periodontitis goes through three clearly established steps: (a) first, a polymicrobial synergy and dysbiosis of the subgingival microbiota, in which the periodontal bacteria with pathogenic potential accumulate, pass through the gingival epithelium, and invade the connective tissues, (b) second, the consequent development of periodontal inflammation in which the host immune response is triggered against the bacterial insult, (c) and finally, periodontal tissue breakdown, which involves the destruction of the connective tissue attachment to the tooth and irreversible alveolar bone resorption [2].

While the presence of periodontal pathogenic bacteria is necessary, it is not sufficient to provoke the onset and progression of the disease [3]. Indeed, inflammation generated by a deregulated immune response plays a key role in disrupting the delicate homeostatic balance between the host and local microbiota. The subgingival microbiota suffers a gradual shift in its composition, from symbiotic to pathogenic bacteria, probably caused by the availability of nutrients coming from the inflammation-derived tissue degradation and the decrease in oxygen concentration as the depth of the periodontal pocket increases [4]. Even though accumulating evidence demonstrates that the exacerbated host immuno-inflammatory response constitutes the primary and ultimate cause of alveolar bone resorption, the cellular mechanisms responsible for the immune dysregulation and overreaction to bacterial challenge remain uncertain [2,3,4,5].

**Figure 1:**
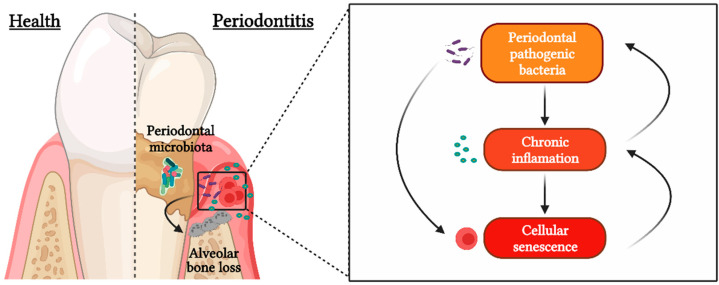
Cellular senescence during periodontitis. Periodontal pathogenic bacteria are capable of causing cellular senescence through the expression of their virulence factors and maintaining a state of chronic inflammation over time, which also induces a favorable nutritional niche for the establishment of these microorganisms and leads to immune dysregulation and consequent tooth-supporting alveolar bone resorption.

In this sense, recent reports suggest that pathologic changes in the periodontitis-affected tissues could create a favorable environment for the induction of cellular senescence, a mechanism that prioritizes cellular survival at the expense of function and that is often accompanied by a pro-inflammatory secretome that contributes to the maintenance of chronic inflammation [6,7]. Cellular senescence is also considered to be the target of some bacterial pathogens, which can subvert some biological processes for their own benefit (Figure 1). For instance, bacteria possess virulence factors capable of inducing cellular senescence responses, either directly by releasing bacterial genotoxins, such as cytolethal distending toxin (CDT), or indirectly by expressing immunogens as lipopolysaccharide (LPS) [8]. In addition to virulence factors, the perpetuation of the inflammation and immune response against bacterial pathogens constitutes another potential way to induce senescence [8].

Curiously, research on the pathophysiology of periodontitis has focused on the recognition of periodontal pathogenic microorganisms and/or their virulence factors that activate the immune system. However, little consideration has been given to cellular senescence in response to the adverse microenvironment created by persistent bacterial infection and chronic inflammation. In this regard, it has been pointed out that states of both persistent infection and chronic inflammation lead to senescence of T lymphocytes, which play a key role in the pathogenesis of periodontitis, and their activity is strongly associated with the severity of its clinical manifestations [9]. Mainly, senescence of CD4^+^ T lymphocytes appears to be a common feature of osteoimmunological diseases, suggesting that CD4^+^ T cell senescence may also occur in the context of periodontitis [7]. This review aims to summarize and discuss the evidence regarding pathogenic senescent CD4^+^ T lymphocytes as a potential mediator in the periodontal microenvironment that contributes to osteoimmunological changes responsible for alveolar bone loss, particularly the Th17/Treg imbalance in periodontitis-affected tissues.

## 2. Cellular Senescence

Cellular senescence is a biological process characterized by the stable arrest of the cell cycle in response to irreversible DNA damage, being a survival alternative to programmed cell death [10]. Replicative senescence was the first type of senescence described. Since all somatic cells have a finite number of cell divisions, known as the Hayflick limit, after each cell division, the length of telomeres (non-coding DNA sequences located at the ends of chromosomes that protect genomic integrity) is shortened. In consequence, there is a point when the telomere shortening is so excessive that it is detected as a DNA double-strand break (DSB), thus triggering cellular senescence in cells subjected to repeated mitotic activity [11]. Interestingly, it was also observed that normal somatic cells subjected to different sources of stress, such as chronic exposure to pro-inflammatory mediators, oxidative stress, or bacterial virulence factors, show features of replicative senescence independent of their telomere length, a phenomenon that was then termed stress-induced cellular senescence [8,11].

**Figure 2:**
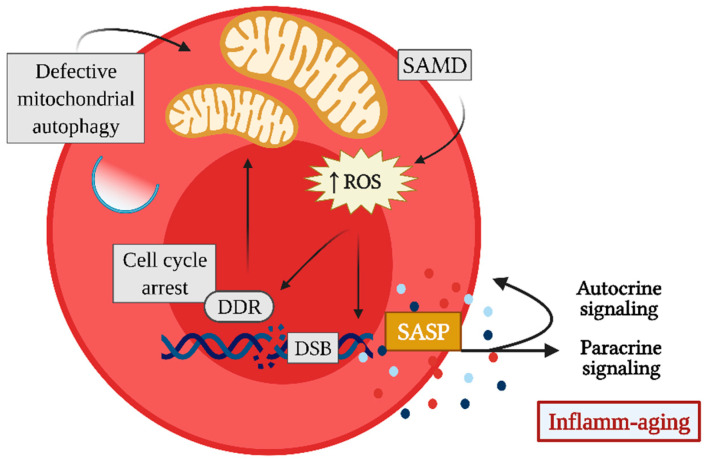
Hallmarks of cellular senescence. Senescence induction can be triggered by multiple stimuli that converge to irreparable DNA damage, such as double-strand breaks (DSB), which activate the DNA damage response (DDR) and lead to cell cycle arrest to prevent replication of the defective cell. Sustained DDR induces senescence-associated mitochondrial dysfunction (SAMD), which together with defective autophagy leads to the accumulation of dysfunctional mitochondria and, consequently, to the increased production of reactive oxygen species (ROS). ROS by themselves are capable, on the one hand, of maintaining DNA damage foci and, on the other hand, of constituting messengers in signal transduction of transcription factors of the senescence-associated secretory phenotype (SASP). Thus, senescent cells produce increased levels of pro-inflammatory cytokines, chemokines, growth factors, and proteases, and some of these secreted molecular mediators contribute to the stabilization of the senescent phenotype in an autocrine manner. At the same time, they also induce senescence in neighboring cells in a paracrine manner.

Senescent cells undergo a phenotypic change, depending on the kind of affected cell and the type, intensity, and duration of the stimulus that caused the DNA damage [10]. However, some phenotypic characteristics appear to be conserved independently of these factors and are frequently used to identify senescent cells (Figure 2). In this context, the main hallmark of cellular senescence is cell cycle arrest, which occurs immediately after damage to the genetic material by the activation of the DNA damage response (DDR), through the recruitment and activation of ataxia-telangiectasia mutated (ATM) and ataxia telangiectasia and Rad3-related protein (ATR) [7,12]. These changes lead to the phosphorylation of H2A histone family member X (γ-H2AX) and the upregulation of cell cycle inhibitors such as p21 and p16^ink4a^ in order to prevent replication of defective cells by blocking their proliferative capacity [7,12].

At a later stage, DDR is capable of triggering senescence-associated mitochondrial dysfunction (SAMD), which is characterized by the presence of large mitochondria with decreased membrane potential and an increased capacity to produce reactive oxygen species (ROS) [13,14]. SAMD is characterized by the production of mitochondrial-derived damage-associated molecular patterns (mito-DAMPs), including cardiolipin, n-formyl peptides, ROS, and mitochondrial DNA, which can be released into the cytoplasm and/or extracellular space and can induce the activation of intracellular signaling pathways, such as NFkB and the NLRP3 inflammasome, resulting in a pro-inflammatory response [15]. In addition, SAMD has a negative effect on the production of ATP, and this can lead to an increase in the AMP/ATP and ADP/ATP ratios and provoke the activation of the AMP-activated protein kinase (AMPK), which senses the reduced energy state during senescence and promotes catabolism while inhibiting biosynthetic pathways [14,16]. Under physiological conditions, dysfunctional mitochondria are recognized and targeted for autophagic digestion for the proper maintenance of cellular function; however, in senescent cells, autophagic and lysosomal functions, necessary for degradation of autophagic vesicle cargo, are compromised, thus preventing the elimination of dysfunctional mitochondria [14,17].

Therefore, the failure of the autophagic process that leads to the accumulation of dysfunctional mitochondria may be responsible for the pro-inflammatory arm of cellular senescence known as the secretory senescence-associated phenotype (SASP), a characteristic of particular biological interest for periodontitis pathogenesis [14,18]. The expression of SASP consists of the secretion of a vast repertoire of pro-inflammatory mediators, such as cytokines, chemokines, growth factors, proteases, and small molecules, including ROS, miRNAs, and extracellular vesicles, which, in some cases, are unique in senescent cells [8,19]. Recently, it has been reported that LPS from the periodontal pathogenic bacteria *Porphyromonas gingivalis* can induce premature senescence in periodontal osteocytes, which adopt an SASP that could further promote alveolar bone resorption [20]. Similarly, *P. gingivalis* can induce premature senescence in dendritic cells by direct cellular invasion, and *P. gingivalis*-induced senescent dendritic cells can acquire an SASP mainly characterized by the secretion of exosomes loaded with inflammasome-related cytokines and age-related miRNAs [21,22].

SASP, unlike DDR and SAMD, does not develop rapidly, but once established, mainly by the initiation of p38 mitogen-activated protein kinase (p38 MAPK) signaling, it persists over time [14,23,24]. In addition, SASP contributes to the stabilization of the senescent phenotype in the cell that expresses it, in an autocrine manner, and induces senescence in neighboring healthy cells, a phenomenon termed paracrine senescence or bystander effect [10,19,25]. Indeed, senescent dendritic cells induced by *P. gingivalis* infection are capable of triggering paracrine senescence in non-senescent dendritic cells after internalization of the SASP-loaded exosomes produced by them, without the need for cell-to-cell contact [21]. Thus, a vicious circle of inflammaging is established, in which SASP components induce senescence, and senescence further contributes to the maintenance of inflammation through SASP [7].

Another characteristic of senescent cells is their resistance to apoptosis. The positive upregulation of senescent cell anti-apoptotic pathways (SCAP) in these cells seems to depend on SAMD, and could explain why they tend to accumulate in tissues in specific pathological contexts [26]. Several prosurvival factors that make senescent cells resistant to apoptosis, such as Bcl-2, protect them from their proapoptotic SASP, although this makes them more sensitive to drugs that interfere with SCAP versus non-senescent cells [16,26,27].

However, it is important to note that there is currently no single reliable marker to identify senescent cells. Therefore, combinations of some hallmarks of the cellular senescence process are used, such as the loss of CD28 expression in the T cell compartment, which has been proposed as a marker of immunosenescence that allows the identification of senescent CD4^+^ T lymphocytes [7].

## 3. Senescent CD4^+^CD28^−^ T Lymphocytes

T lymphocytes constitute a main cellular component of the adaptive arm of the immune response. Their activation involves the recognition of an antigen, presented via the major histocompatibility complex (MHC) by an antigen-presenting cell (APC) through their T-cell receptor (TCR) [28,29]. However, the TCR/MHC-antigen interaction is not a sufficient synaptic stimulus to activate T lymphocytes; it requires the joint engagement of CD28, a costimulatory molecule receptor expressed on the T-cell surface that binds to CD80 (B7.1) and CD86 (B7.2), expressed by the APCs [28,29]. Thus, the expression of CD28 is necessary for the induction and activation of antigen-mediated immune responses in T lymphocytes [29].

Interestingly, the loss of CD28 expression is the most consistent biomarker of immunosenescence [30]. In T lymphocytes, the loss of CD28 occurs through the depletion of a CD28-specific initiator complex, including the nuclear proteins nucleolin and heterogeneous nuclear ribonucleoprotein (hnRNP)-D0, and results in its transcriptional silencing [31]. The presence of CD28^−^ or CD28^null^ T lymphocytes has been observed in replicative senescence and stress-induced senescence, as well as in normal chronological aging, and in tissues with chronic inflammation and affected by persistent infection [7,9].

Notably, the senescence of T lymphocytes under inflammatory conditions and in persistent infections can be attributed to the following factors. After T lymphocyte activation, extensive cell proliferation occurs, termed clonal expansion, which gives rise to T lymphocytes with identical antigen-specific TCRs [29]. However, repeated antigenic stimulation and a lack of resolution of the immune response could result in the oligoclonal expansion of T lymphocytes with an already high replication history and can potentially lead to replicative senescence. Although there is a vast repertoire of TCR during infections, only a small number of T lymphocytes have TCRs capable of recognizing and responding to the presence of specific microorganism-derived antigens [32,33]. In this context, it has been reported that repeated antigenic stimulation of these T-cell clones leads to the irreversible loss of CD28, accompanied by telomere shortening due to decreased telomerase activity [34]. T lymphocytes senescence can also be induced by genotoxic stress, in particular, inflammatory mediators such as interferon (IFN)-α, tumor necrosis factor (TNF)-α, prostaglandin E2 (PGE2), and ROS can induce the loss of CD28 and a senescent cellular phenotype [35,36,37,38]. To date, it is not known whether periodontal pathogenic bacteria and their virulence factors can generate senescence in T lymphocytes that infiltrate periodontitis-affected tissues.

**Figure 3:**
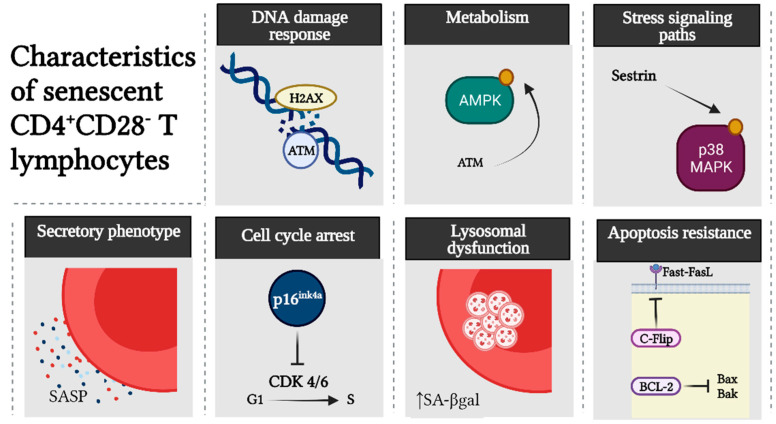
Hallmarks of senescent CD4^+^CD28^−^ T lymphocytes. In contrast to their non-senescent counterpart, senescent CD4^+^CD28^−^ T lymphocytes show activation of the DNA damage response (DDR) by ataxia-telangiectasia mutated (ATM) and phosphorylation of H2A histone family member X (γ-H2AX). ATM signaling in these cells leads to phosphorylation of AMP-activated protein kinase (AMPK) and subsequently through a sestrin-mediated signaling pathway to p38 MAPK. Senescent CD4^+^CD28^−^ T lymphocytes also show increased secretion of pro-inflammatory and cytotoxic mediators attributed to a senescence-associated secretory phenotype (SASP) and upregulated cell cycle inhibitors, such as p16^ink4a^, that block cell cycle progression from the G1 to the S phase. In addition, they have lysosomal dysfunction, as evidenced by increased senescence-associated-β-galactosidase (SA-βgal) activity, and are highly resistant to apoptosis by upregulating anti-apoptotic proteins such as Bcl-2 and cellular Flice-inhibitory protein (c-Flip).

CD4^+^CD28^−^ T lymphocytes show classic hallmarks of cellular senescence (Figure 3). Senescent CD4^+^CD28^−^ T lymphocytes, in comparison with their non-senescent CD4^+^CD28^+^ counterparts, show increased expression of DDR markers such as ATM, which downstream phosphorylates γ-H2AX and AMPK [39,40]. In turn, AMPK can maintain constitutive p38 MAPK activation, leading to inhibition of the telomerase and signalosome components essential for the TCR signaling pathway [39,40]. It is noteworthy that loss of CD28 or signalosome components does not imply a state of anergy or exhaustion in senescent T cells; on the contrary, they show an increased capacity to produce pro-inflammatory cytokines and cytotoxic mediators as part of their SASP [7,41]. The acquisition of these properties appears to be independent of TCR signaling and dependent on the overexpression of innate immunity receptors, such as toll-like receptors (TLRs) and natural killer cell lectin-like receptor G1 (KLRG1), which can also be complementarily used for the identification of senescent T lymphocytes [41,42]. Other features of cellular senescence observed in CD4^+^CD28^−^ T lymphocytes are the up-regulation of p16^ink4a^, lysosomal dysfunction detected by increased senescence-associated-β-galactosidase (SA-βgal) activity, and high resistance to apoptosis through both the intrinsic and extrinsic pathways, due to the overexpression of the anti-apoptotic proteins Bcl-2 and cellular Flice-inhibitory protein (c-Flip), respectively [43,44,45,46].

Therefore, senescent CD4^+^CD28^−^ T lymphocytes are considered oligoclonal, dysfunctional, and terminally differentiated. Although they tend to accumulate over time and are shown in more significant numbers in the elderly than young people, they can also be generated prematurely, particularly in pathological contexts, where they can contribute to immune abnormalities related to the disease [7,9].

## 4. Osteoimmunology and Th17/Treg Imbalance

**Figure 4:**
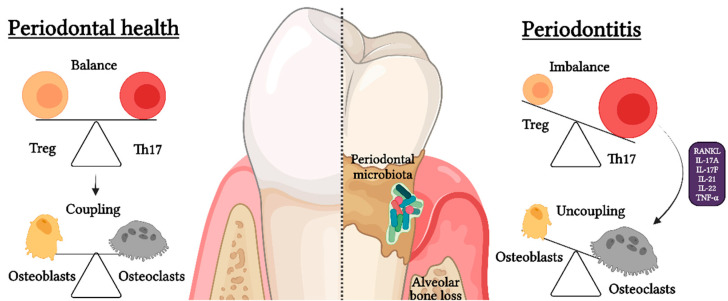
Th17/Treg imbalance during periodontitis. During periodontal health, there is a balance between the Th17- and Treg-pattern of the immune response, which determines the molecularly mediated cellular coupling between osteoclasts and osteoblasts and thus the consequent physiological process of bone remodeling. However, during periodontitis, there is a Th17/Treg imbalance that favors the Th17-pattern of immune response and leads to osteoclast/osteoblast uncoupling. Remarkably, this Th17/Treg imbalance provokes an increased differentiation and activation of osteoclast caused by the production of elevated levels of pro-osteoclastogenic mediators, such as receptor–activator of NF-κB ligand (RANKL), interleukin (IL)-17A, IL-17F, IL-21, IL-22, and tumor necrosis factor (TNF)-α, which finally lead to irreversible tooth-supporting alveolar bone loss.

Osteoimmunology, which studies the direct interaction between the immune system and bone, has recently gained prominence in research on inflammatory bone loss during periodontitis. In this regard, it has been consistently established that CD4^+^ T lymphocyte activity, specifically the balance between Th17 and Treg cell activities, directly influences osteoclastogenesis and osteoclast/osteoblast coupling regulation [47]. Under physiological conditions of bone remodeling, there is a cellular coupling between osteoclast and osteoblast activities, in which the bone resorption process is followed by the process of bone formation to maintain bone volume [48]. However, under pathological conditions such as periodontitis, the rate of bone resorption/formation is higher due to osteoclast/osteoblast uncoupling, which responds in part to an increase in osteoclastic activity favored by the Th17/Treg imbalance (Figure 4) [2,47].

Th17 lymphocytes express the following master-switch transcription factors: signal transducer and activator of transcription (STAT)-3 and retinoic acid receptor-related orphan nuclear receptor-γt (RORγt) RORC2 in humans [49,50]. Th17 lymphocytes have been described as a subset of CD4^+^ T cells with the capacity to induce osteoclast differentiation and activation during the pathogenesis of periodontitis [49,50,51]. Indeed, Th17 lymphocytes are characterized by the secretion of pro-inflammatory cytokines, such as IL-17A, IL-17F, IL-21, IL-22, and TNF-α, which can induce the production of receptor–activator of NF-κB ligand (RANKL) by fibroblasts and osteoblasts [52,53,54]. Th17 lymphocytes can also express RANKL by themselves, thereby producing and amplifying the RANKL signaling [49,55,56]. RANKL, binding to its specific receptor RANK expressed on the surface of osteoclast precursors, induces a signaling cascade that terminates in the activation of NF-κB and NFATc1, the master regulators of osteoclast differentiation [57]. Subsequently, they activate specific bone resorption genes, such as cathepsin K and tartrate-resistant acid phosphatase (TRAP), essential for osteoclast activity [57]. On the other hand, IL-17A and TNF-α may synergize with RANKL by provoking the increase of RANK expression in osteoclast precursors, thus leading to enhanced sensitivity to RANKL even at suboptimal concentrations [58,59]. Moreover, IL-17A, IL-21, IL-22, and TNF-α are able to induce TRAP^+^ osteoclasts in the absence of exogenous RANKL in vitro, although the intracellular signaling pathways involved remain unknown [60,61]. With respect to osteoblasts, TNF-α and IL-17A are able to inhibit osteoblastogenesis by inducing the upregulation of Dickkopf (Dkk)-1, which in turn inhibits the Wnt-β-catenin-signaling necessary for differentiation of stromal mesenchymal cells into osteoblasts [7,62]. IL-17A has the ability to downregulate osteoblast genes associated with bone formation, such as alkaline phosphatase (ALP), osteocalcin (OCN), and Runt-related transcription factor 2 (Runx-2), and even cause pyroptosis in already differentiated osteoblasts [63,64].

As a functional cellular counterpart, Tregs express the master-switch transcription factors STAT-5 and forkhead box-p3 (Foxp3) and high levels of CD25 [65]. Tregs are a subset of CD4^+^ T cells that exhibit an opposite function to Th17 lymphocytes by being able to protect against inflammation and alveolar bone loss during periodontitis [66]. Functionally, Tregs are characterized by the secretion of immunoregulatory cytokines, such as IL-4, IL-10, IL-35, and transforming growth factor (TGF)-β, which regulate and suppress the inflammatory response, including that of Th17 lymphocytes [66]. Other common mechanisms of immune regulation include the regulation of APC activity through the expression of cytotoxic T-lymphocyte antigen-4 (CTLA-4) and metabolic exhaustion of pro-inflammatory Th1 and Th17 lymphocytes by the local consumption of IL-2 [66]. In relation to differentiation and activation of osteoclasts, it has been shown that Tregs are able to inhibit monocyte/macrophage differentiation into osteoclasts through their anti-inflammatory cytokines and CTLA-4 activity, which induces the activation of the enzyme indoleamine 2,3-dioxygenase (IDO) in osteoclast precursors and promotes their apoptosis [67,68]. With respect to osteoblasts, Treg function in some contexts is necessary for osteoblast differentiation by triggering the Wnt10b production by other T-cell subsets, which induce the activation of Wnt-β-catenin signaling for the differentiation of stromal mesenchymal cells into osteoblasts [69,70]. Interestingly, recent reports have suggested that the Treg phenotypic instability during periodontitis contributes to even more bone loss because these unstable Tregs produce more RANKL than Th17 lymphocytes [71,72].

Under physiological conditions, the balance between Th17 and Treg activities allows the dynamic regulation of bone homeostasis by maintaining osteoclast/osteoblast coupling; however, a Th17/Treg imbalance occurs during periodontitis, leading to osteoclast/osteoblast uncoupling and promotion of bone resorption. The cause behind the Th17/Treg imbalance during periodontitis has not been fully elucidated.

## 5. Senescent T Lymphocytes and Th17/Treg Imbalance during Periodontitis

The periodontitis-affected tissue microenvironment provides an ideal niche for the induction of cellular senescence [6]. Indeed, periodontal pathogenic bacteria may serve as persistent antigenic stimuli while being a stress factor for periodontal cells [7]. In addition, the local increase of pro-inflammatory mediators such as cytokines, ROS, and PGE2 during periodontitis may constitute factors capable of inducing a senescent phenotype in T lymphocytes [7].

**Figure 5:**
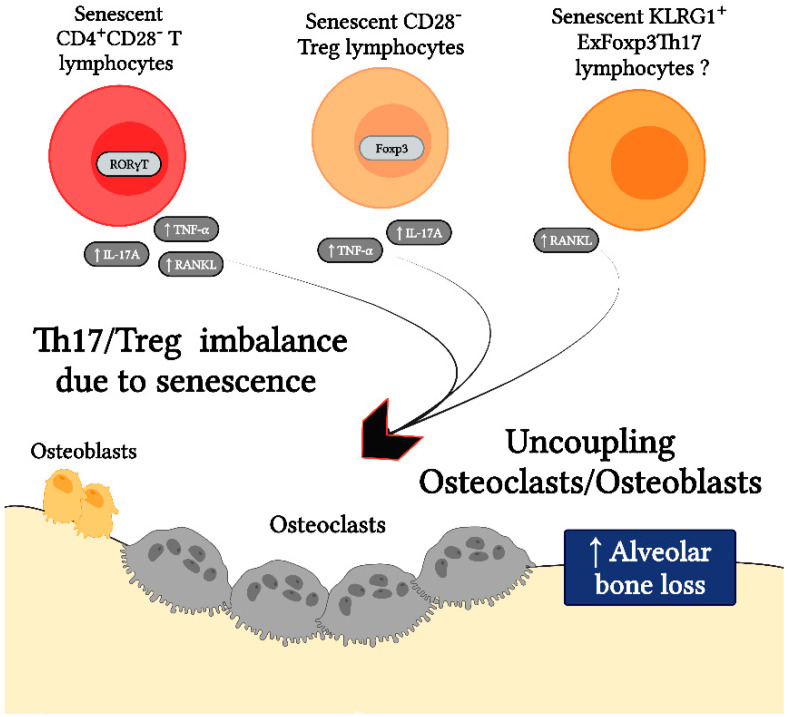
Senescence in the CD4^+^ T cell compartment leads to Th17/Treg imbalance that can trigger osteoclast/osteoblast uncoupling and alveolar bone loss. Senescent CD4^+^CD28^−^ T lymphocytes are biased to Th17-like polarization and function, which is evidenced by their increased expression of the transcription factor retinoic acid receptor-related orphan nuclear receptor-γt (RORγt), the bone resorptive factor receptor activator of NF-κB ligand (RANKL), and the cytokines interleukin (IL)-17A and tumor necrosis factor (TNF)-α, presumably as part of their senescence-associated secretory phenotype (SASP). Similarly, senescent Foxp3^+^CD4^+^CD28^−^ Tregs show features of phenotypic instability, such as lower suppressive capacity and production of IL-17A and TNF-α. Furthermore, Tregs that lose Foxp3 expression and express the signature senescent marker killer cell lectin-like receptor G1 (KLRG1) also produce higher amounts of RANKL. All of these senescent T cells can lead to osteoclast/osteoblast uncoupling in favor of osteoclast differentiation and activation, resulting in increased tooth-supporting alveolar bone loss.

In this context, patients affected by other diseases distinct from periodontitis and similar to periodontitis, characterized by persistent infection and chronic inflammation, have a disproportionately high number of CD28^−^ T lymphocytes representing senescent T cells in comparison with the CD28^+^ counterpart and, in some cases, these T-cell levels are associated with the severity of clinical manifestations [9]. In these diseases, it is noteworthy that senescent CD4^+^CD28^−^ T lymphocytes have been associated with a Th17-type immune response and loss of phenotypic stability of the Treg profile, resulting in increased osteoclastogenesis and consequent bone loss (Figure 5). Indeed, CD4^+^CD28^−^ T lymphocytes isolated from rheumatoid arthritis patients show a preferential polarization towards the Th17 phenotype with expression of the transcription factor RORγt [73]. Senescent CD4^+^CD28^−^ T lymphocytes also show higher RANKL and IL-17A expression levels and increased induction of TRAP^+^ osteoclasts when compared to their non-senescent CD4^+^CD28^+^ T lymphocyte counterpart [74,75]. Similarly, CD4^+^CD28^−^ T lymphocytes generated during osteoporosis express higher levels of TNF-α and have the capacity to induce TRAP with greater intensity in osteoclasts compared to CD4^+^CD28^+^ T lymphocytes [76].

Indeed, accumulating evidence shows that senescent CD4^+^CD28^−^ T lymphocytes are a common feature of chronic osteolytic pathologies such as rheumatoid arthritis, osteopenia, osteoporosis, and osteomyelitis and during the loss of orthopedic bone implants due to infectious causes [74,77,78,79]. Although these cells have not been described in periodontitis, it seems to share similar osteoimmunological bone resorption phenomena with other osteolytic diseases, despite having different etiologies. In this regard, several studies show that periodontitis-affected patients possess leukocytes with significantly shorter telomeres than age-matched healthy subjects, and these levels associate with disease severity, suggesting replicative senescence in these cells [80,81]. Therefore, it could be suggested that senescent CD4^+^CD28^−^ T lymphocytes may play an important role in the pathogenesis of alveolar bone loss characteristic of periodontitis.

Similar to effector CD4^+^ T lymphocytes, Tregs can also undergo cellular senescence. Senescent Foxp3^+^CD4^+^CD28^−^ T lymphocytes have been identified in patients with rheumatoid arthritis, which downregulate CD25 while showing increased expression levels of TNF-α and IL-17A in addition to decreased suppressive capacity [44]. These Foxp3^+^CD4^+^CD28^−^ Tregs also showed enhanced expression of senescence markers, such as increased SA-βgal activity and DDR when compared to Foxp3^+^CD4^+^CD28^+^ T lymphocytes [44]. Interestingly, the inflammatory milieu present in both rheumatoid arthritis and periodontitis-affected tissues is able to provoke Treg phenotypic instability, substantially downregulating their Foxp3 and CD25 expression, the hallmarks of their regulatory phenotype [71,82]. These cells also show extensively decreased immunosuppressive capacity and acquire the ability to produce Th17-type cytokines, such as RANKL and IL-17A, having an enhanced osteoclastogenic capacity compared to conventional Th17 lymphocytes [71,82]. Interestingly, exFoxp3 Tregs express the signature senescence marker KLRG1, which may represent senescent CD4^+^ T lymphocytes; however, further studies are needed to characterize them in detail [71,82]. Taken together, these data allow us to suggest that senescence within the periodontitis-related CD4^+^ T lymphocyte compartment may partly explain the Th17/Treg imbalance that results in osteoclast/osteoblast uncoupling and favors alveolar bone resorption. Accordingly, Table 1 summarizes the evidence supporting the possible role of CD4^+^CD28^−^ T cell senescence as a potential driver of Th17/Treg imbalance during periodontitis.

## 6. p38 MAPK as a Driver of SASP Development in Senescent CD4^+^CD28^−^ T Cells

**Figure 6:**
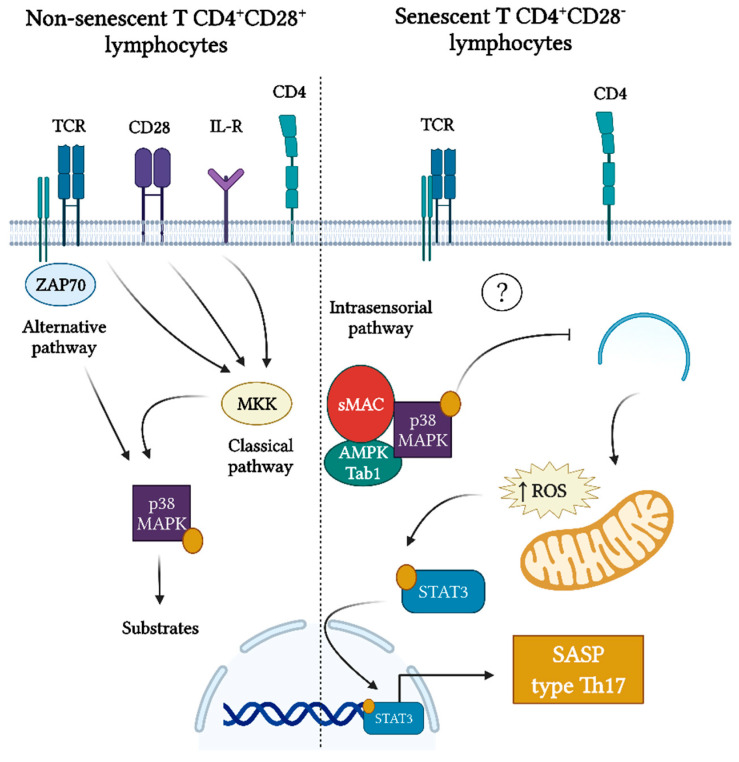
Activation of p38 MAPK in senescent CD4^+^CD28^−^ T lymphocytes and its potential to drive a Th17-type SASP. Activation of p38 mitogen-activated protein kinase (p38 MAPK) in non-senescent CD4^+^ T lymphocytes can respond to two signaling pathways: classical and alternative. The classical signaling pathway can be activated by cytokines or by engagement of the T cell receptor (TCR) and the costimulatory molecule CD28 and depends on an upstream kinase cascade that relies on mitogen-activated protein kinase kinase (MAPKK) activation. In contrast, the alternative signaling pathway is activated by TCR engagement and depends on zeta chain-associated protein kinase 70 (ZAP70). However, senescent CD4^+^CD28^−^ T lymphocytes lack MAPKK and ZAP70 but show constitutive p38 activation, which responds to an intracellular sensory mechanism in response to DNA damage and is dependent on the AMP-activated protein kinase (AMPK)-Tab1 and sestrin-MAPK activation complex (sMAC). It has been observed that p38 MAPK is able to inhibit autophagy and lead to the accumulation of dysfunctional mitochondria, with the consequent increase of reactive oxygen species (ROS). In CD4^+^ T lymphocytes of elderly individuals, it has been shown that this process leads to the activation of the signal transducer and activator of transcription (STAT)-3, responsible for the production of a Th17 profile of cytokines.

The activation of p38 MAPK plays a critical role in inflammation. Indeed, it has been strongly associated with human periodontal inflammation and severity [84]. Apart from that, it has been demonstrated that p38 MAPK activity is key to SASP production in different types of senescence, including replicative senescence and stress-induced senescence; in fact, the blockade of its activity inhibits SASP expression [23,24]. In CD4^+^ T lymphocytes, p38 MAPK is activated by phosphorylation of the 180-threonine and 182-tyrosine residues and depends on two signaling pathways: classical or canonical and alternative [88]. The classical signaling pathway is triggered by cytokines or by the engagement of the costimulatory receptor CD28 and the TCR, which depends on a kinase cascade culminating in the activation of mitogen-activated protein kinase kinase (MAPKK). In contrast, the alternative signaling pathway is triggered exclusively by the engagement of the TCR, which induces p38 autophosphorylation and depends on zeta chain-associated protein kinase 70 (ZAP70) [88]. Interestingly, senescent CD4^+^CD28^−^ T lymphocytes show constitutive phosphorylation of p38 MAPK that does not depend on the traditionally described classical or alternative pathways, but an intracellular sensory mechanism in response to DNA damage and is dependent on sestrin-MAPK activation complex (sMAC) [39,40] (Figure 6). Even so, activation of p38 MAPK by itself can lead to senescence in non-senescent CD4^+^ T lymphocytes [39].

In senescent CD8^+^ T lymphocytes, activation of p38 MAPK leads to the inhibition of autophagy by the down-regulation of p38IP-mAtg9 interaction and results in the accumulation of dysfunctional mitochondria and increased mitochondrial ROS [89]. Interestingly, CD4^+^ T lymphocytes from older individuals show diminished autophagy, accumulation of dysfunctional mitochondria, and increased ROS, leading to the phosphorylation of STAT-3 and consequently, to the production of Th17-related mediators that could trigger differentiation and activation of osteoclasts and inhibition of osteoblasts [87] (Figure 6). Recently, it was reported that CDT genotoxin, a virulence factor present in pathogenic Gram-negative bacteria closely linked to periodontitis, is able to induce senescence in CD4^+^ T lymphocytes [83]. Moreover, DDR signaling can trigger the p38 MAPK activation and consequently, the production of a distinct SASP pattern in activated CD4^+^ T lymphocytes, including the production of Th17-type cytokines [83]. However, it is unknown whether the activation of p38 MAPK in senescent CD4^+^CD28^−^ T lymphocytes is responsible for the autophagic dysfunction that could eventually induce the production of a Th17-biased SASP; thus, more studies on this subject are necessary.

## 7. Autophagic Dysfunction during Senescence and Th17/Treg Imbalance

Autophagy is a conserved physiological process that plays a key role in the Th17/Treg balance, and senescent cells show drastic changes in terms of the autophagic function that impact mitochondrial homeostasis. Th17 lymphocytes are the functional phenotype most resistant to autophagic blockade [90]. In contrast, this subset of T lymphocytes is highly sensitive to autophagy induction. In fact, differentiated Th17 lymphocytes treated with rapamycin, metformin, or polyamine spermidine significantly reduce the percentage of CD4^+^IL-17^+^ T lymphocytes and lead to an increase in Foxp3 expression through a mechanism that promotes autophagic activity [91,92,93]. Conversely, Tregs are the T-cell functional phenotype most dependent on autophagy. Inhibition of autophagy in Tregs leads to altered cellular energy metabolism, increased predisposition to apoptosis, increased production of IFN-γ and IL-17A, and even increased susceptibility to Foxp3 expression loss [94,95]. These reports suggest that defective autophagy in senescent CD4^+^CD28^−^ T cells may partly contribute to the loss of Th17/Treg balance during cellular senescence.

Defective autophagy in senescent CD4^+^ T lymphocytes can modulate the Th17/Treg imbalance through metabolic reprogramming [96,97]. For instance, glycolysis has been established as critical during Th17 cell development and the production of their cytokines [96]. Besides, it has been reported that during T-cell differentiation, Th17-inducing cytokines drive glycolysis by mTORC1 signaling, which in turn limits the metabolic supply for *N*-glycan branching, as a requirement for T-cell differentiation [97]. On the other hand, naïve T lymphocytes under Th17-polarized conditions enhance the production and activity of glycolytic enzymes, whereas the blockade of glycolysis leads to inhibition of Th17 differentiation and promotion of Treg differentiation [97]. Indeed, already differentiated Th17 lymphocytes also depend on glycolysis to carry out their function [96,98]. In fact, mTORC1 has downstream targets that positively regulate STAT-3, HIF-1α, S6K1, and S6K2 activation, which through different pathways lead to the production of Th17-type cytokines [96,98].

In Tregs, autophagy favors mitochondrial OXPHOS and restricts glycolysis by inhibiting mTORC1 activity [94]. In this context, Treg metabolism is primarily supported by OXPHOS, which is reflected in a higher mitochondrial mass in this type of cell, in comparison with effector CD4^+^ T lymphocytes, and is essential for their suppressive function [91,99,100]. In turn, Foxp3 expression contributes to blocking mTORC1 signaling, in order to increase OXPHOS while decreasing glycolysis [100]. Recently, it was reported that the alteration of mitochondrial complex III, a key enzyme complex in OXPHOS, is essential for the maintenance of the suppressive functions in active Tregs [98]. Interestingly, the specific ablation of this complex in Tregs leads to the development of a lethal inflammatory disease, without altering the Treg number or Foxp3 expression; instead, the cells showed a decreased OXPHOS activity and an increased glycolytic flux [98]. Indeed, OXPHOS has been shown to lead to the increased synthesis of downstream branched *N*-glycan, a post-translational modification that regulates the levels of cell surface immunosuppressive proteins during Treg differentiation, including CD25, GITR, PD-1, PD-L1, CD73, CTLA-4, and ICOS [101,102].

Thus, autophagic dysfunction during cell senescence could be a mechanism that contributes, on the one hand, to Treg lineage instability by inducing SAMD, impairing OXPHOS, and favoring the glycolytic pathway, and on the other hand, to Th17 lymphocyte differentiation and function, by modulating mTORC1 activity. It should also be noted that, although it is known that T17 and Treg lymphocytes can transdifferentiate in some conditions, senescent CD4^+^CD28^−^ T lymphocytes are defined as terminally differentiated cells, a phenomenon that may imply a lineage commitment biased to Th17-like immune response and loss of cellular plasticity [103,104]. These phenomena can compromise the immune response and regulation against perpetual antigenic challenge, possibly contributing to chronic periodontitis inflammation.

## 8. Towards the Encounter of Periodontitis-Related Senescent T Lymphocytes

Cellular senescence of T lymphocytes in host-pathogen interactions and inflammatory osteolysis is an emerging field, particularly in the context of periodontitis. Although in vitro studies contribute to closing knowledge gaps, several questions need to be addressed in in vivo and ex vivo models, including the identification of senescent T lymphocytes in the periodontal microenvironment, the detailed analysis of their produced SASP, their role in the periodontitis pathogenesis, as well as the molecular regulatory mechanisms that would be involved. We propose some experimental designs to address some of these questions:

*Detection of senescent CD4^+^ T lymphocytes in periodontitis-affected tissues and characterization of their SASP:* In periodontal tissues, senescent CD4^+^ T lymphocytes can be identified by quantifying T cell-specific senescence markers using immunofluorescence and/or flow cytometry in experimental animal models of periodontitis and comparing their detection in healthy controls. This technological proposal further allows isolation of this particular cell population by fluorescence-activated cell sorting for in situ and ex vivo analyses, such as single-cell sequencing or mass spectrometry, in order to achieve a detailed characterization of their SASP components.

*Role of the SASP produced by senescent CD4^+^ T lymphocytes in periodontitis-associated alveolar bone resorption:* To elucidate the potential role of senescent CD4^+^ T lymphocytes in the pathogenesis of periodontitis, an adoptive transfer model can be used in immunodeficient mice lacking mature T cells. In this experimental model, a purified population of senescent or non-senescent CD4^+^ T lymphocytes can be transferred and periodontitis can be induced in these mice. Then, the presence of TRAP^+^ osteoclasts by histochemistry and alveolar bone loss by micro-computed tomography can be analyzed, thus determining the osteoclastogenic and pro-resorptive potential of these cells in vivo.

*SASP regulatory mechanisms in senescent CD4^+^ T lymphocytes:* To determine the molecular mechanisms involved in SASP production by senescent CD4^+^ T lymphocytes purified from periodontitis-affected tissues, it is possible to use p38 MAPK, ROS, and/or autophagy inhibitors and evaluate their influence on the production of pro-inflammatory mediators, thus elucidating the main regulators orchestrating SASP production in these cells.

## 9. T Lymphocyte Senescence as a Potential Therapeutic Approach during Periodontitis

In general terms, senotherapeutic approaches are aimed at eliminating selectively senescent cells through the use of senolytic strategies or at reversing negative aspects of senescence, such as SASP, through senomorphic strategies [12]. Senolytic strategies do not appear to have long-term negative consequences and have been shown to have beneficial effects on bone during aging by decreasing bone loss and maintaining bone formation. However, this approach has not been evaluated in senescent CD4^+^ T lymphocytes in the context of diseases with inflammatory osteolysis, such as periodontitis [12,105].

The discovery of some mechanisms of SASP regulation has allowed the creation of novel strategies for its modulation, such as the inhibition of p38 MAPK signaling and the use of metformin [12]. The inhibition of p38 MAPK in senescent CD4^+^ T lymphocytes significantly enhanced telomerase activity and cell survival after TCR activation [89,106]. While in senescent CD8^+^ T lymphocytes, it increased autophagic activity and mitochondrial function, in addition to the downregulation of TNF-α expression [89,106]. On the other hand, the use of metformin rescued autophagy and mitochondrial function in CD4^+^ T lymphocytes in the elderly, leading to decreased ROS production and downregulation of the Th17-associated cytokines IL-6, IL-17A, and IL-21, which could decrease the periodontal osteoclastogenic stimuli [87].

In the context of periodontitis, preclinical studies have shown that inhibition of p38 MAPK prevents bone loss by decreasing osteoclast formation and downregulation of pro-inflammatory cytokines, such as IL-1β, IL-6, and TNF-α [85,86]. Similarly, preclinical and clinical studies using metformin have shown effectiveness in reducing bone loss and improving periodontal clinical outcomes by decreasing the RANKL/OPG ratio [107]. In this context, it remains to be determined whether CD4^+^CD28^−^ T lymphocytes play a pathological role during the development of periodontitis as suggested by the emerging literature, which may contribute to the understanding of the pathophysiological process of the disease and the development of new therapeutic approaches.

## 10. Concluding Remarks

Persistent infection and chronic inflammation during periodontitis perfectly maintain the perpetual local antigenic stimuli and inflammatory milieu ideal for the induction of cellular senescence. Indeed, the chronically inflamed periodontitis-affected tissues enriched in pro-inflammatory mediators favor the continuance of a deregulated immune response characterized by the Th17/Treg imbalance that leads to pathologic alveolar bone resorption. This inflammatory niche is sufficient to provoke the loss of the CD28 marker and the establishment of SASP expression on CD4^+^ T lymphocytes. Moreover, the characteristic Th17-biased immune response and loss of Treg immunoregulatory capacities given by senescent T cells certainly match the periodontitis immune tone. Different strategies of cell senescence therapeutic regulation seem to effectively rescue autophagy and mitochondrial activity from senescence and downregulate inflammatory mediators and alveolar bone loss from periodontitis. Therefore, up-to-date accumulated evidence suggests that senescence in CD4^+^ T lymphocytes could play a critical role during the pathogenesis of periodontitis.

## Figures and Tables

**Table 1:** Evidence supporting the possible role of CD4^+^CD28^−^ T cell senescence as a potential driver of Th17/Treg imbalance during periodontitis.

Evidence	Findings	References	Evidence Suggests That
Association of periodontitis with leukocyte telomere length.	Patients with periodontitis show leukocytes with significantly shorter telomeres than age-matched healthy subjects, which is associated with disease severity.	[80,81]	Periodontitis causes early replicative senescence in leukocytes.
Pro-inflammatory mediators induce T lymphocyte senescence.	Inflammatory mediators, such as interferon (IFN)-α, tumor necrosis factor (TNF)-α, prostaglandin E2 (PGE2), and ROS, are able to induce CD28 loss and senescence of T lymphocytes in vitro.	[35,36,37,38]	These mediators, being present in periodontitis, may trigger senescence of CD4^+^ T lymphocytes.
Bacterial genotoxins induce CD4^+^ T lymphocyte senescence.	The cytolethal distending toxin (CDT), a virulence factor present in the Gram-negative bacterium *Aggregatibacter actinomycetemcomitans* closely related to periodontitis etiology, induces premature senescence in CD4^+^ T lymphocytes in vitro and in vivo models. In this context, it is assumed that the produced SASP is induced by the activation of p38 MAPK signaling.	[83]	Periodontal pathogenic bacteria may play an important role in the induction of senescence in CD4^+^ T lymphocytes.
Differential activation of p38 MAPK signaling during periodontitis.	The phospho-p38 MAPK intensity score in immunostained tissues was positively correlated with clinical periodontal parameters of the disease linked to inflammation and bone loss, implying that p38 MAPK activation is one of the main signaling pathways involved in human periodontal inflammation and its severity. Inhibition of p38 MAPK activation in preclinical models of periodontitis prevented bone loss.	[84,85,86]	There could be a relation between the activation of the p38 MAPK signaling pathway in senescent CD4^+^ T lymphocytes and SASP production.
Senescent CD4^+^ T lymphocytes exhibit a Th17-biased secretory profile.	Senescent CD4^+^CD28^−^ T lymphocytes show a preferential polarization towards the Th17 phenotype, with the increased expression of RORγt. In addition, CD4^+^ T lymphocytes from elderly subjects show a Th17-biased cytokine production profile, due to the defects in autophagy and mitochondrial bioenergetics, which in turn are associated with redox imbalance and activation of the Th17 master regulator STAT-3 to bind to IL-17A promoters.	[73,87]	The senescence of CD4^+^ T lymphocytes during periodontitis may favor the Th17 lymphocyte polarization.
Senescent CD4^+^ T lymphocytes are a common feature of chronic osteolytic pathologies.	Senescent CD4^+^CD28^−^ T lymphocytes are present in chronic osteolytic pathologies such as rheumatoid arthritis, osteopenia, osteoporosis, osteomyelitis, and during the loss of orthopedic bone implants due to infectious causes. In these contexts, senescent CD4^+^CD28^−^ T lymphocytes show a greater osteoclastogenic capacity due to a higher production of RANKL and TNF-α, as compared with their non-senescent counterparts.	[74,77,78,79]	Senescent CD4^+^ T lymphocytes may be directly linked to alveolar bone loss due to the increased production of pro-osteoclastogenic mediators.
Senescent Tregs show impaired suppressor function and increased production of pro-inflammatory profile cytokines.	A novel subset of senescent CD28^−^ Treg is described, which insufficiently suppressed the proliferation of effector T lymphocytes and produced a pro-inflammatory cytokine pattern.	[44]	Senescent Tregs in peridontitis may be related to an imbalance between their regulatory and effector functions.
During experimental periodontitis, exFoxp3Th17 KLRG1^+^ lymphocytes are generated.	During experimental periodontitis, Foxp3^+^ T lymphocytes are converted into exFoxp3Th17 cells, expressing KLRG1. KLRG1 is a hallmark of cellular senescence in T lymphocytes. Thus, exFoxp3Th17 cells play a key role in the pathogenesis of periodontitis by expressing high amounts of IL-17A and RANKL and showing a potent osteoclastogenic capacity in vivo.	[71]	Senescent Tregs appear to be generated in the context of periodontitis and play a key role during the alveolar bone resorption due to the polarization bias towards the Th17 phenotype.

## Data Availability

Not applicable.
